# The Impacts of a Psychoeducational Alcohol Resource During Internet-Delivered Cognitive Behavioral Therapy for Depression and Anxiety: Observational Study

**DOI:** 10.2196/44722

**Published:** 2023-04-18

**Authors:** Vanessa Peynenburg, Ram P Sapkota, Tristen Lozinski, Christopher Sundström, Andrew Wilhelms, Nickolai Titov, Blake Dear, Heather Hadjistavropoulos

**Affiliations:** 1 University of Regina Regina, SK Canada; 2 Karolinska Institutet Stockholm Health Care Services Stockholm Sweden; 3 Macquarie University Sydney Australia

**Keywords:** internet-delivered cognitive behavioral therapy, transdiagnostic, depression, anxiety, alcohol, drinking

## Abstract

**Background:**

Problematic alcohol use is common among clients seeking transdiagnostic internet-delivered cognitive behavioral therapy (ICBT) for depression or anxiety but is not often addressed in these treatment programs. The benefits of offering clients a psychoeducational resource focused on alcohol use during ICBT for depression or anxiety are unknown.

**Objective:**

This observational study aimed to elucidate the impacts of addressing comorbid alcohol use in ICBT for depression and anxiety.

**Methods:**

All patients (N=1333) who started an 8-week transdiagnostic ICBT course for depression and anxiety received access to a resource containing information, worksheets, and strategies for reducing alcohol use, including psychoeducation, reasons for change, identifying risk situations, goal setting, replacing drinking with positive activities, and information on relapse prevention. We assessed clients’ use and perceptions of the resource; client characteristics associated with reviewing the resource; and whether reviewing the resource was associated with decreases in clients’ alcohol use, depression, and anxiety at posttreatment and 3-month follow-up among clients dichotomized into *low-risk* and *hazardous* drinking categories based on pretreatment Alcohol Use Disorders Identification Test (AUDIT) scores.

**Results:**

During the 8-week course, 10.8% (144/1333) of clients reviewed the resource, and those who reviewed the resource provided positive feedback (eg, 127/144, 88.2% of resource reviewers found it worth their time). Furthermore, 18.15% (242/1333) of clients exhibited hazardous drinking, with 14.9% (36/242) of these clients reviewing the resources. Compared with nonreviewers, resource reviewers were typically older (*P*=.004) and separated, divorced, or widowed (*P*<.001). Reviewers also consumed more weekly drinks (*P*<.001), scored higher on the AUDIT (*P*<.001), and were more likely to exhibit hazardous drinking (*P*<.001). Regardless of their drinking level (ie, low risk vs hazardous), all clients showed a reduction in AUDIT-Consumption scores (*P*=.004), depression (*P*<.001), and anxiety (*P*<.001) over time; in contrast, there was no change in clients’ drinks per week over time (*P*=.81). Reviewing alcohol resources did not predict changes in AUDIT-Consumption scores or drinks per week.

**Conclusions:**

Overall, ICBT appeared to be associated with a reduction in alcohol consumption scores, but this reduction was not greater among alcohol resource reviewers. Although there was some evidence that the resource was more likely to be used by clients with greater alcohol-related difficulties, the results suggest that further attention should be given to ensuring that those who could benefit from the resource review it to adequately assess the benefits of the resource.

## Introduction

### Background

Over the last few decades, internet-delivered cognitive behavioral therapy (ICBT) has been established as an effective treatment option for a variety of mental health concerns [1]. The content of ICBT often mirrors that of face-to-face cognitive behavioral therapy (eg, cognitive restructuring, behavioral activation, breathing strategies, and relapse prevention) but is offered in a web-based format, often in the form of a course. In particular, transdiagnostic ICBT has been developed to reduce clients’ needs to engage in multiple courses of therapy to address comorbidities [2] and has been found to be similarly effective to disorder-specific treatment programs in some studies [3,4].

In transdiagnostic ICBT for depression or anxiety, it is relatively uncommon to address comorbid alcohol use difficulties. This represents a missed opportunity, as previous research suggests that heavy drinking days are common among ICBT clients, with 56.8% (514/905) of clients endorsing drinking ≥6 drinks on 1 occasion during the past year [5]. Moreover, in another study on ICBT for depression, panic disorder, and social anxiety, 24.1% (381/1581) of clients reported drinking difficulties [6]. Interestingly, alcohol use difficulties have not been found to predict ICBT completion or outcomes in terms of improved depression or anxiety, which supports the use of ICBT for depression and anxiety among individuals with comorbid alcohol use difficulties [5]. The extent to which transdiagnostic ICBT is associated with reduced alcohol use or problems over time remains unknown.

In the literature, we identified only 1 study in which participants completing a self-guided ICBT program for depression received a brief intervention related to alcohol use [7]. In this study, participants were randomized to receive either a self-guided ICBT program for depression (MoodGYM; Australian National University) or the same program with a normative feedback intervention for alcohol use (Check Your Drinking). The Check Your Drinking screener was administered at baseline, and participants received a report summarizing their drinking compared with others of the same age, sex, and country (ie, Canada), which could be accessed at any point while clients completed the ICBT program [7]. Adding a brief feedback intervention did not predict drinking or depression outcomes. However, the Check Your Drinking intervention did not provide strategies for managing alcohol use. Thus, more research is needed to understand how alcohol-related treatment may be beneficial in ICBT for depression and anxiety.

### Objectives

This study included an evaluation of data from clients enrolled in an ICBT course offered in a Canadian province (Saskatchewan) over a span of 1 year (January to December 2021). This observational study aimed to add to the sparse literature and explore whether an additional alcohol resource available to clients at any point during transdiagnostic ICBT would be used and positively evaluated by clients. Furthermore, this study sought to explore whether alcohol use improved over time among clients in ICBT and, more specifically, among those who reviewed resources with low-risk or hazardous alcohol consumption.

We aimed to explore the following research questions: (1) What percentage of clients review the alcohol resource? (2) What client characteristics are associated with reviewing the alcohol resource? (3) Is the use of transdiagnostic ICBT generally associated with improvements in alcohol consumption over time? (4) Compared with clients who do not review the alcohol resource, do those who review resources show greater improvements in alcohol consumption, depression, and anxiety over time than those who do not? and (5) How will clients who review the alcohol resource evaluate it?

Given the limited nature of previous research in this area and that this was an exploratory analysis, the only hypothesis was that clients endorsing alcohol use problems at pretreatment would be more likely to review alcohol resources.

## Methods

### Design

This study was an uncontrolled observational trial conducted within the Online Therapy Unit, which is a Saskatchewan government–funded ICBT clinic that accepts clients for treatment on an ongoing basis. This study included data from the Online Therapy Unit’s regular service delivery.

### Ethics Approval

The study was approved by the institutional research ethics board of the University of Regina (approval number 2019-197).

### Participants

#### Recruitment

Prospective clients learned about the services of the Online Therapy Unit through a variety of sources (ie, family physicians, other medical professionals, community mental health clinics, web-based searches, word of mouth, media, and posters or cards).

#### Sample Size

This study included all clients (N=1333) who started ICBT for anxiety and depression at the Online Therapy Unit during 2021, allowing for the analysis of a full year of service delivery.

#### Eligibility Criteria

**Figure 1:**
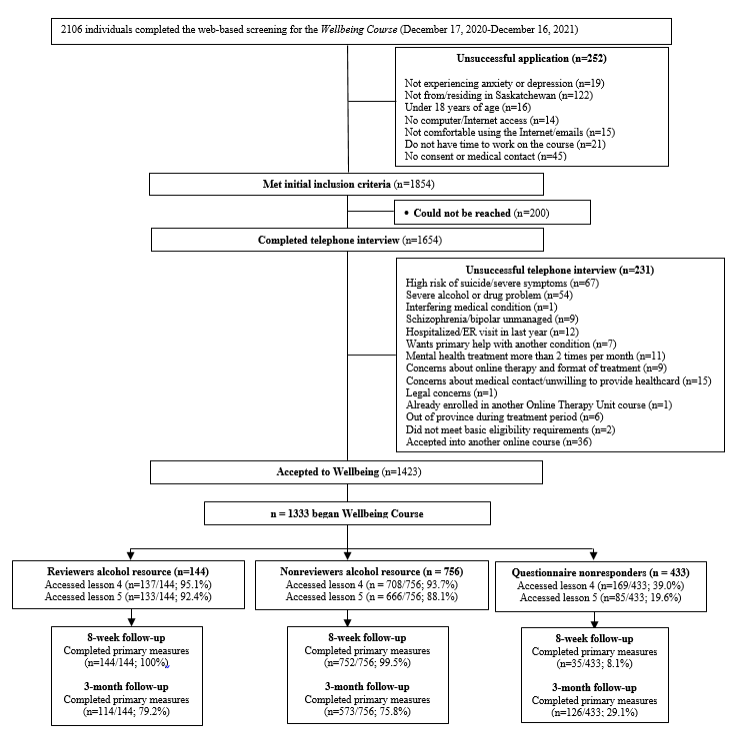
Client flow from screening to 3-month follow-up. ER: emergency room.

All clients first completed a web-based screening via the Online Therapy Unit website, after which a telephone screening call was made. Prospective clients were eligible for the Online Therapy Unit’s services and to be a part of this study if they endorsed (1) being aged at least 18 years, (2) experiencing a minimum of mild depression or anxiety symptoms as their primary concern or concerns, (3) residing in Saskatchewan for the duration of treatment, (4) having access to and comfort using a computer and the internet, (5) a willingness to provide emergency medical contact (eg, family physician and psychiatrist), and (6) consenting to and beginning ICBT. Furthermore, prospective clients were excluded from this study if they reported or were assessed as (1) exhibiting unmanaged psychosis or mania, (2) demonstrating high suicide risk, (3) receiving mental health support from another provider more than twice per month, and (4) experiencing severe difficulties with alcohol use (ie, scoring ≥20 on the Alcohol Use Disorder Identification Test [AUDIT]) [8] or other substance use (ie, scoring ≥25 on the Drug Use Disorder Identification Test [DUDIT]) [9]. Figure 1 shows a client flowchart.

### Measures

#### Overview

All primary and secondary outcome measures were administered at pretreatment, posttreatment (8 weeks after enrollment), and 3-month follow-up. Clients completed treatment satisfaction and resource evaluation questions at posttreatment. Although clients responded to additional questions about their symptoms over the course of the 8-week treatment period, the measures listed below were the focus of this study. Clients also completed questionnaires assessing insomnia, panic, social anxiety, mental health-related disability, treatment experiences, mental health service use, and pandemic-related anxiety but were not used as part of this observational study.

#### Baseline Screening Measures

##### AUDIT Screening Tool

AUDIT [8] is a well-standardized 10-item screening measure for alcohol use difficulties. The total scores range from 0 to 40, with higher scores indicating greater alcohol-related difficulties. Scores from 6 to 14 (for women) and 8 to 14 (for men) indicate hazardous alcohol use, scores from 15 to 19 indicate harmful alcohol use, and scores ≥20 indicate possible alcohol dependence [10]. In this trial, we referred to all scores ≥6 (for women) and ≥8 (for men or other) as indicative of hazardous alcohol use. In this study, the Cronbach α for the AUDIT was .75.

##### DUDIT Screening Tool

The DUDIT [9] is a well-standardized 11-item screening tool for substance use difficulties. The total scores ranged from 0 to 44. Higher scores indicated greater difficulties in substance use. Scores from 2 to 24 (for women) and 6 to 24 (for men) indicated difficulties with substance use, and scores ≥25 indicated possible substance dependence. In this study, the Cronbach α for the DUDIT was .77.

##### Demographics

Clients responded to questions regarding their age, gender, relationship status, race and ethnicity, location, education, and employment status during the web-based screening. Furthermore, clients were asked if they had taken psychotropic medications within the past 3 months.

#### Primary Outcome Measures

##### AUDIT-Consumption

AUDIT-Consumption (AUDIT-C) [11] consists of the first 3 consumption items of the full AUDIT [12]. Previous studies have suggested that the AUDIT-C has similar sensitivity and specificity indices as the full AUDIT [13]. Cronbach α for the AUDIT-C in this study was .62 and .77.

##### Total Drinks in Previous Week

Clients were asked to indicate how many standard drinks of alcohol they had drank in the last 7 days. This question is commonly used in ICBT trials of alcohol misuse [14,15].

#### Secondary Outcome Measures

##### Patient Health Questionnaire 9-Item

The Patient Health Questionnaire 9-item (PHQ-9) [16] is a screening measure for depression, with total scores ranging from 0 to 27. Scores <5 indicated minimal depression, and scores ≥10 were used to identify probable cases of major depressive disorder [17]. Cronbach α in this study was between .84 and .89.

##### Generalized Anxiety Disorder 7-Item

The Generalized Anxiety Disorder 7-item (GAD-7) [18] is a measure to screen for generalized anxiety disorders. Total scores ranged from 0 to 21, with scores <5 indicating minimal symptoms of anxiety and scores ≥10 indicating clinically significant symptoms of generalized anxiety [18]. Cronbach α in this study was between .87 and .91.

##### Treatment Engagement

Clients’ treatment engagement was captured based on whether they completed all 5 lessons as well as their total number of website log-ins over the 8-week treatment period. The platform does not track how long clients are logged in because this is a biased estimate impacted by whether clients fail to log out.

##### Treatment Satisfaction

Clients were asked yes-or-no questions about whether the course was worth their time and whether they would recommend the course to a friend. On 5-point scales, they were also asked to rate their overall satisfaction with the course (1=*very dissatisfied* to 5=*very satisfied*).

##### Alcohol Resource Evaluation Survey

Clients were asked a yes-or-no question about whether they reviewed the alcohol resource. If they reported reviewing the resource, clients were asked to rate their level of effort dedicated to reviewing the resource from 1=*none at all* to 7=*a great deal*. Furthermore, from 1=*not at all* to 7=*very*, clients were asked (1) how understandable the resource was, (2) if they learned something new from the resource, and (3) how helpful the resource was. Clients then responded to 3 open-ended questions asking them to describe what they liked about the resource, what they disliked about the resource, and the nature of any changes they made to their drinking because of reviewing the resource.

### Intervention

#### The Well-being Course

All clients were offered a therapist-assisted well-being course, which is an 8-week transdiagnostic ICBT course for depression and anxiety [3]. The course was developed at the eCentreClinic at Macquarie University and is licensed for use by the Online Therapy Unit. During the course, clients read five web-based lessons based on key components of cognitive behavioral therapy: (1) psychoeducation about symptoms and the cognitive behavioral model; (2) thought monitoring and challenging; (3) physical symptoms of depression or anxiety, de-arousal strategies, and pleasant activity scheduling; (4) graded exposure; and (5) relapse prevention. Clients read materials presented as slides, case stories, frequently asked questions, and downloadable guides, with homework activities and lesson summaries. All materials were presented in the English language. Lessons were released gradually over 8 weeks, and clients needed to complete each lesson before proceeding to the next lesson. Clients received automated reminder emails as upcoming lessons became available. Furthermore, all clients were assigned to a therapist for the duration of the 8-week ICBT program. Clients received either optional therapist support (ie, support provided at client requests) or once-weekly support. These 2 approaches for providing therapist support in ICBT have been found to be effective [19].

In addition to the 5 core lessons, clients can access additional downloadable resources at any time. The resources addressed a wide range of topics, namely, anger, alcohol use, assertiveness, beliefs, chronic conditions, chronic pain, communication skills, grief, health anxiety, mental skills, motivation, new motherhood, panic, posttraumatic stress disorder, sleep, workplace mental health, and worry. In this trial, therapists informed clients about the availability of resources during their first message to clients and made tailored recommendations based on the clients’ presenting concerns. Clients can also self-select the resources to review. During week 5, therapists asked all clients whether they had questions about any additional resources that they had reviewed.

#### Alcohol Resource

The alcohol resource was based on content included in an ICBT program for alcohol use called the Alcohol Change Course [14], which was developed in collaboration with a patient-oriented research steering committee comprising researchers, clinicians, stakeholders, trainees, and patient partners with lived experiences. The Alcohol Change Course and the alcohol resource use a relapse prevention model to address alcohol use [20]. The resource consisted of 17 pages and was in a downloadable format. Content wise, the resource started with a section on the relationship between alcohol use and mental health and provided information about the stimulating and inhibiting effects of alcohol, reasons for drinking alcohol, how alcohol use difficulties can vary in severity, and Canada’s low-risk drinking guidelines (enacted in 2011) that recommended consuming ≤10 (for women) and ≤15 (for men) weekly drinks [21]. The next sections focused on how alcohol affects physical health; the connection between alcohol consumption, depression, and anxiety; and the impact of alcohol on sleep. The remaining sections highlighted strategies for changing one’s drinking habits. Clients were prompted to consider their reasons for drinking and were provided worksheets to note the pros and cons of drinking, how drinking does or does not align with their values, and their reasons for change. A list of questions was provided to assist the clients in setting goals related to alcohol use. Thereafter, the resource included sections on identifying personal strengths and supports, changing the availability of alcohol in one’s day-to-day life, a worksheet on identifying risk situations and positive activities to replace drinking, information on “slips,” and a summary of the resource.

#### Therapist Support

Clients who scored in the clinical range (≥10) on either the PHQ-9 or GAD-7 were offered optional or regular once-weekly therapist support. Clients who scored in the nonclinical range (<10) on both PHQ-9 and GAD-7 were offered optional weekly support. In regular once-weekly therapist support, therapists reviewed symptom measures and sent clients a brief, tailored message once a week. Clients could also be contacted via phone if they had not logged in during the past week, if their PHQ-9 or GAD-7 scores increased by 5 or more points, or if there was an indication of elevated suicide risk. In optional support, therapists would only contact clients if they initiated contact that week, if their PHQ-9 or GAD-7 score increased by 5 or more points, or if there was an indication of elevated suicide risk. Previous research has shown that both approaches are similarly effective, and approximately 25% of clients prefer optional support [19,22].

### Data Analyses

#### Overview

Analyses were conducted using SPSS (version 28.0.0.0; IBM Corp) [23]. In an initial review of the data, the following 3 client groups were identified: clients who reported reviewing the alcohol resource (“reviewers”; n=144), clients who reported not reviewing the alcohol resource (“nonreviewers”; n=756), and those who did not respond to the Alcohol Resource Evaluation Survey (“questionnaire nonresponders” [QNRs]; n=433). Descriptive statistics were used to describe the pretreatment characteristics of the 3 groups. Furthermore, ANOVA, *χ*^2^ analyses, and *t* tests (2-tailed) were used to assess group differences on all pretreatment variables. A significance level of *P*=.01 was used as a partial control for multiple comparisons.

There were no missing data for the clients’ baseline screening variables. Data for the primary and secondary outcome variables were missing mainly because of client dropout (eg, 4/896, 0.4% to 213/687, 23.7% at posttreatment and follow-up, respectively). The missing data were determined to be missing completely at random (MCAR) via Little MCAR test (*χ*^2^_44_=56.8; *P*=.09) [24].

To assess whether there was a significant change in clients’ AUDIT-C scores and previous weekly drinks over time, as well as to determine if pretreatment, posttreatment, and follow-up scores differed across clients and between groups (ie, reviewers vs nonreviewers), a series of mixed models were computed using the maximum likelihood estimation method with 3 assessment points (ie, pretreatment, posttreatment, and follow-up). As only 900 clients reported whether they reviewed the alcohol resource, mixed model analyses were performed with this subsample (*group*=reviewers vs nonreviewers) to assess the rate of change in these clients’ AUDIT-C scores and previous weekly drinks as well as their depression and anxiety. These analyses were also conducted with clients dichotomized based on their pretreatment AUDIT scores into *low-risk* drinking (ie, scores <6 for women and <8 for men or other) and *hazardous* drinking (ie, scores ≥6 for women and ≥8 for men or other).

Missing data were not imputed because the data were assumed to be MCAR, and linear mixed model analysis can handle missing data [25]. Fixed effects for *time*, *group*, and their interactions (ie, *time* × *group*) and random effects for the intercept and *time* variables were tested and included in the model to account for the correlated nature of the data. Intraclass correlation coefficients were calculated to identify the proportion of variance across clients and to determine if mixed model analyses were appropriate [26]. To select the model that best fit the data, various within-individual and between-individual covariance structures (eg, scaled identity, diagonal, unstructured, and autoregressive) were tested. Models with the smallest Akaike information criterion and Bayesian information criterion were retained for the final analysis. The repeated-measure indicator variable, *time*, was recoded as 0, 2, and 5 to reflect the actual assessment months (ie, 0=pretreatment, 2=posttreatment at 2 months [8 weeks], and 5=follow-up at 5 months).

#### Treatment Engagement and Satisfaction

Treatment engagement and satisfaction were compared between alcohol resource reviewers and nonreviewers through a combination of ANOVA and *χ*^2^ analyses.

#### Qualitative Data Analysis

Client responses to the Alcohol Resource Evaluation Survey were analyzed using conventional qualitative content analysis [27]. The literature on including a resource for managing alcohol use in transdiagnostic ICBT is limited; therefore, conventional qualitative content analysis can be helpful for identifying quantifiable response categories [28].

The coding process consisted of the following steps:

1. VP reviewed all client responses to questions regarding what clients liked about the alcohol resource, disliked about the resource, and any changes they made to their drinking because of the resource. A codebook was created during the review. The codebook consisted of identified codes, a description of each code, and an example quote for each code. A decision to review all client responses to ensure saturation was made during the creation of the codebook.
2. AW and TL used the codebook to independently code all client responses. The 2 coders were able to assign more than 1 code to a client response where appropriate (eg, if clients identified making more than 1 change after reviewing the resources).
3. VP reviewed the responses of AW and TL to identify and resolve instances of disagreement.

## Results

### Client Characteristics

The pretreatment client characteristics of the overall sample are presented in Table 1. Most clients reported identifying as a woman (1036/1333, 77.72%), being in a married or common law relationship (1210/1333, 90.77%), identifying as White (1158/1333, 86.87%), living in a large city (778/1333, 58.36%), being educated beyond high school (1058/1333, 79.37%), and being employed either part time or full time (699/1333, 52.44%). In terms of clinical characteristics, more than half of the clients (732/1333, 54.91%) reported taking psychotropic medication or medications in the past 3 months, and most of the sample exhibited clinically remarkable symptoms of depression (955/1333, 71.64%) and anxiety (949/1333, 71.19%). The rates of alcohol consumption per week and hazardous alcohol use are summarized in Table 1. Of note, while 49.67% (662/1333) of the clients did not consume any previous weekly drinks at pretreatment, 6.8% (45/662) of these clients reviewed the alcohol resource. Similarly, 24.46% (326/1333) of clients reported never drinking alcohol by scoring 0 on the pretreatment AUDIT; however, 5.8% (19/326) of these clients reviewed the alcohol resource.

Table 1 displays the pretreatment characteristics of resource reviewers, nonreviewers, and QNRs. In terms of significant differences between resource reviewers and nonreviewers, resource reviewers were older (*t*_898_=2.90; *P*=.004), more likely to be men (*χ^2^*_1_=16.2; *P*<.001), as well as more often separated, divorced, or widowed (*χ*^2^_2_=14.2; *P*<.001). Furthermore, compared with nonreviewers, reviewers consumed more weekly drinks (*t*_898_=6.08; *P*<.001); scored significantly higher on the AUDIT (*t*_898_=6.81; *P*<.001); and were more likely to score above the cutoff, indicating hazardous drinking (*χ*^2^_1_=14.6; *P*<.001). Most clients received standard once-weekly therapist support (reviewers: 84/144, 58.3%; and nonreviewers: 448/756, 59.2%).

In terms of significant differences between reviewers and QNRs, QNRs were typically younger (*t*_575_=7.81; *P*<.001), were more often single or never married (*χ*^2^_2_=20.3; *P*<.001), and had a lower education level compared with resource reviewers (*χ*^2^_2_=11.1; *P*=.004). In addition, QNRs were more likely to be women (*χ*^2^_1_=7.9; *P*=.005), consumed fewer weekly drinks (*t*_575_=4.11; *P*<.001), and had higher depression symptoms (*t*_575_=3.14; *P*=.002).

**Table 1:** Client characteristics at pretreatment.

Variable					All clients (N=1333)			Reviewers (n=144)			Nonreviewers (n=756)			Questionnaire nonresponders (n=433)			Significance					
																	Test				*P* value	
Age (years), mean (SD; range)					38.04 (13.78; 18-86)			43.17 (13.70; 20-77)			39.46 (14.16; 18-86)			33.85 (11.95; 18-77)			*F*_2,1330_=35.79				<.001	
**Gender, n (%)**																		*χ*^2^_2_=16.2				<.001
	Woman			1036 (77.72)			94 (65.3)			608 (80.4)			334 (77.1)									
	Man or other			297 (22.28)			50 (34.7)			148 (19.6)			99 (22.9)									
**Relationship status, n (%)**																		*χ*^2^_4_=34.7				<.001
	Single or never married			408 (30.61)			29 (20.1)			211 (27.9)			168 (38.8)									
	Married or common law			802 (60.17)			90 (62.5)			485 (64.2)			227 (52.4)									
	Separated, divorced, or widowed			123 (9.22)			25 (17.4)			60 (7.9)			38 (8.8)									
**Ethnicity, n (%)**																		*χ*^2^_4_=5.8				.22
	Indigenous			81 (6.08)			9 (6.2)			38 (5)			34 (7.8)									
	Other			94 (7)			9 (6.2)			49 (6.5)			36 (8.3)									
	White			1158 (86.87)			126 (87.5)			669 (88.5)			363 (83.8)									
**Location, n (%)**																		*χ*^2^_4_=6.7				.15
	Large city (>100,000)			778 (58.36)			89 (61.8)			437 (57.8)			252 (58.2)									
	Small to medium city			190 (14.25)			13 (9)			104 (13.8)			73 (16.8)									
	Small rural location (<7000)			365 (27.38)			42 (29.2)			215 (28.4)			108 (24.9)									
**Education, n (%)**																		*χ*^2^_4_=23.5				<.001
	High school or less			275 (20.63)			20 (13.9)			143 (18.9)			112 (25.9)									
	More than high school or less than university			589 (44.19)			69 (47.9)			317 (41.9)			203 (46.9)									
	University education			469 (35.18)			55 (38.2)			296 (39.2)			118 (27.2)									
**Employment status, n (%)**																		*χ*^2^_4_=9.8				.04
	Employed part time or full time			699 (52.44)			75 (52.1)			380 (50.3)			244 (56.4)									
	Unemployed or disability			250 (18.75)			27 (18.8)			135 (17.8)			88 (20.3)									
	Homemaker, student, or retired			384 (28.81)			42 (29.2)			241 (31.9)			101 (23.3)									
**Pretreatment scores**																						
	**Value, n (%)**																					
		Psychotropic medication in the past 3 months	732 (54.91)			77 (53.5)			405 (53.6)			250 (57.7)			*χ*^2^_2_=2.1				.36			
		AUDIT^a^ ≥6 for women and ≥8 for men or other	242 (18.15)			36 (25)			96 (12.7)			110 (25.4)			*χ*^2^_2_=35.0				<.001			
		PHQ-9^b^ ≥10	955 (71.64)			102 (70.8)			506 (66.9)			347 (80.1)			*χ*^2^_2_=23.7				<.001			
		GAD-7^c^ ≥10	949 (71.19)			104 (72.2)			512 (67.7)			333 (76.9)			*χ*^2^_2_=11.4				.003			
	**Value, mean (SD)**																					
		Drinks per week	2.89 (6.78)			6.35 (15.63)			2.23 (4.42)			2.87 (4.69)			*F*_2,1330_=23.00				<.001			
		AUDIT	3.39 (3.76)			4.75 (4.17)			2.72 (3.07)			4.11 (4.11)			*F*_2,1330_=30.37				<.001			
		PHQ-9	13.50 (5.74)			13.34 (5.61)			12.65 (5.69)			15.03 (5.58)			*F*_2,1330_=24.48				<.001			
		GAD-7	12.64 (5.09)			12.51 (5.00)			12.10 (5.06)			13.61 (5.04)			*F*_2,1330_=12.23				<.001			

^a^AUDIT: Alcohol Use Disorders Identification Test.

^b^PHQ-9: Patient Health Questionnaire 9-item.

^c^GAD-7: Generalized Anxiety Disorder 7-item.

### Primary Outcome Variables

Clients’ mean AUDIT-C scores were 2.33 (SD 2.09) at pretreatment, 2.22 (SD 1.94) at posttreatment, and 2.11 (SD 1.94) at 3-month follow-up. The mixed model analysis predicting clients’ AUDIT-C scores revealed a significant decrease in AUDIT-C scores over time (β=−.03, SE 0.01; *P*=.004). Although not significant, there was a negative correlation (*r*=−0.17; *P*=.06) between the intercept and slope, which may indicate that compared with those with lower pretreatment AUDIT-C scores, individuals with higher scores experienced greater reductions in scores over time. Furthermore, although there was a significant *group* (ie, reviewers vs nonreviewers) effect (β=1.10, SE 0.171; *P*<.001), the interaction effect was not significant (*P*=.69). This indicates that although reviewers and nonreviewers varied significantly in pretreatment AUDIT-C scores, reviewing the alcohol resource did not influence changes in their AUDIT-C scores over time. The subsequent mixed model analysis, with clients dichotomized into low-risk and hazardous drinking subgroups, showed a significant decrease in AUDIT-C scores over time for clients in the hazardous drinking subgroup (β=−.21, SE 0.06; *P*=.002). In contrast, *time* was not significant for clients in the low-risk subgroup (*P*=.14). Although there was a significant *group* effect for clients in the low-risk subgroup (β=.74, SE 0.16; *P*<.001), there was no *group* effect for those in the hazardous subgroup (*P*=.91). Furthermore, there was no significant interaction effect for either the low-risk (*P*=.44) or hazardous (*P*=.83) drinking subgroups, indicating that reviewing alcohol resources had no effect on the rate of change in clients’ AUDIT-C scores over time, irrespective of drinking problems.

On average, clients consumed 2.89 (SD 6.78) weekly drinks at pretreatment, 2.44 (SD 4.18) weekly drinks at posttreatment, and 2.77 (SD 5.29) weekly drinks at 3-month follow-up. The mixed model analysis predicting clients’ previous weekly drinking showed that there was no significant *time* effect (*P*=.81) or interaction effect (*P*=.35). Furthermore, the mixed model analyses dichotomizing clients into low-risk and hazardous drinking subgroups showed no reductions in weekly drinks over time for both clients in the low-risk (*P*=.24) and hazardous (β=−.80, SE 0.40; *P*=.05) drinking subgroups. There was no significant interaction effect for either the low-risk (*P*=.84) or hazardous (*P*=.66) drinking subgroups.

In sum, the results of the primary analyses show that regardless of their pretreatment alcohol use difficulties, there was a significant decrease in clients’ AUDIT-C scores over time and no change in clients’ previous weekly drinks over time. Furthermore, reviewing the alcohol resources did not influence changes over time in clients’ AUDIT-C scores or previous weekly drinks.

### Secondary Outcome Variables

#### Overview

Clients’ mean PHQ-9 scores were 13.50 (SD 5.75) at pretreatment, 6.85 (SD 5.36) at posttreatment, and 5.90 (SD 4.97) at 3-month follow-up. Furthermore, their mean GAD-7 scores were 12.64 (SD 5.09) at pretreatment, 6.16 (SD 4.98) at posttreatment, and 5.36 (SD 4.83) at 3-month follow-up. Mixed model analyses revealed significant *time* effects for depression (β=−1.10, SE 0.04; *P*<.001) and anxiety (β=−1.22, SE 0.04; *P*<.001). Yet, there was no significant main effect (*P*=.45) or interaction effect (*P*=.34) of *group* (ie, reviewers vs nonreviewers) predicting depression. Furthermore, although there was no main effect (*P*=.53) of *group* predicting decreases in anxiety, there was a significant interaction effect (β=−.21, SE 0.10; *P*=.04), indicating that the anxiety of alcohol resource reviewers decreased more than that of nonreviewers.

Moreover, the mixed model analysis dichotomizing clients into low-risk and hazardous drinking subgroups showed a statistically significant decrease in depression over time among clients in both the low-risk (β=−1.35, SE 0.05; *P*<.001) and hazardous (β=−1.58, SE 0.19; *P*<.001) drinking subgroups. There were no statistically significant *group* or interaction effects for either the low-risk (group: *P*=.19; interaction: *P*=.18) or hazardous (group: *P*=.17; interaction: *P*=.73) drinking subgroup. These findings suggest that reviewing alcohol resources had no effect on changes in clients’ PHQ-9 scores over time, irrespective of their level of drinking.

Similarly, the mixed model analysis dichotomizing clients into low-risk and hazardous drinking subgroups revealed a significant decrease in anxiety over time for clients in both the low-risk (β=−1.20, SE 0.04; *P*<.001) and hazardous (β=−1.41, SE 0.15; *P*<.001) drinking subgroups. There was no significant *group* effect for clients in either the low-risk (*P*=.27) or hazardous (*P*=.13) drinking subgroup. Although the interaction effect was significant in the low-risk subgroup (β=−.26, SE 0.12; *P*=.03), it was not significant in the hazardous subgroup (*P*=.54). These results suggest that for clients in the low-risk drinking group, resource reviewers’ anxiety decreased more than that of nonreviewers.

#### Treatment Engagement and Satisfaction

Treatment engagement was also examined by resource reviewers and nonreviewers (n=900; Table 2). Treatment completion rates were high in both groups, with 88.8% (799/900) of the clients accessing all 5 ICBT lessons. No significant group differences were found for any measure of treatment engagement or satisfaction (all *P*>.01).

**Table 2:** Treatment engagement and satisfaction.

Variable			Reviewers and nonreviewers (n=900)		Reviewers (n=144)		Nonreviewers (n=756)		Significance		
									Test		*P* value
**Engagement**											
	Accessed lesson 5, n (%)	799 (88.8)		133 (92.4)		666 (88.1)		*χ*^2^_1_=2.2		.14	
	Number of website log-ins, mean (SD)	26.55 (27.78)		26.58 (15.44)		26.54 (29.56)		*t*_898_=.01		.99	
**Satisfaction, n (%)**											
	Course was worth the time	846 (96)		140 (97.2)		724 (95.8)		*χ*^2^_1_=.7		.41	
	Would recommend course to friend	867 (96.3)		140 (97.2)		727 (96.2)		*χ*^2^_1_=.4		.54	
	Satisfied or very satisfied overall	744 (82.7)		124 (86.1)		620 (82)		*χ*^2^_1_=1.4		.23	

#### Evaluation of Alcohol Resource

A total of 144 clients responded to the Alcohol Resource Evaluation Survey. Clients indicated dedicating a moderate amount of effort into the resource (mean 3.79, SD 1.92), moderately agreed that they had learned something new by reviewing the resource (mean 4.31, SD 2.00), found the resource moderately helpful (mean 4.74, SD 1.78), and rated the resource as very understandable (mean 6.15, SD 1.11). Most clients who reviewed the resources indicated that it was worth their time (127/144, 88.2%).

#### Likes About the Resource

Of the 144 responses, 122 (84.7%) were codable as “likes” (see Table 3 for codes). The most common liked aspect of the alcohol resource was that it was informative (eg, “It was informative and I was able to learn information I wasn’t aware of before” [client ID 35854]). This was followed by comments about how the resource gave clients insight into their drinking (eg, “It made me evaluate alcohol use” [client ID 34723]). Some clients also commented on how they liked the way the information was presented (eg, “easy to read and understand, not complicated at all” [client ID 35645]). The remaining comments focused on how clients appreciated learning about the relationship between alcohol consumption and symptoms of depression and anxiety (eg, “It helped me see alcohol in a different way. It helped me see the effects it was implementing on my anxiety.” [client ID 35902]), how they were able to use the resource to either better understand or support their loved ones’ drinking difficulties (eg, “I was able to understand some things and pass it along to my husband to use” [client ID 35876]), how the resource acted as a review of information they had learned about alcohol use in the past (eg, “It was consistent with and validated other resources I have encountered over the years”), how they liked specific worksheet activities included in the resource (eg, “I like that it gave a worksheet to list the values of why I wanted to reduce drinking” [client ID 36683]), and how they appreciated the information provided about the negative effects of drinking (eg, “It explained a lot about the effects of alcohol on the physical and mental components of the body” [client ID 36029]).

**Table 3:** Client responses to the Alcohol Resource Evaluation Survey.

Responses				Example		Client ID		Values, n (%)	
**What did you like about the resource? (n=144)**									
	Informative			“Learning things I didn’t know.”		35687		62 (43.1)	
	Not a like			“I do not drink. Therefore doesn’t pertain to me.”		35755		23 (16)	
	Insight into one’s drinking			“Helped me to gain more perspective about my drinking and helped me make the commitment to stop drinking completely”		34424		21 (14.6)	
	Presentation of information			“Very clear and concise”		36081		18 (12.5)	
	Information on the relationship between alcohol use and symptoms of anxiety or depression			“It confirmed for me the effects alcohol can have on depression and mood.”		35688		12 (8.3)	
	Resource allowed them to help or understand others			“I used the resource to help me with family members who are alcohol users”		35722		10 (6.9)	
	Resource acted as a refresher			“it refreshed some knowledge I had”		36292		8 (5.6)	
	Worksheet activity			“The question prompts were helpful to think about.”		36027		7 (4.9)	
	Information on the negative effects of drinking			“That there are negative consequences beyond just drinking too much.”		35829		6 (4.2)	
**What did you not like about the resource? (n=144)**									
	Nothing			“There was not anything I didn’t like.”		34368		109 (75.7)	
	Not relevant to client experience			“It did not apply to me; I drink a couple beers a year.”		34054		12 (8.3)	
	Format or structure issues			“I wish the PDF was fillable”		35890		7 (4.9)	
	Insight into one’s drinking			“I honestly don’t remember anything I didn’t like except maybe how it forced me to think more actively about my drinking/alcohol consumption patterns.”		36643		5 (3.5)	
	No new information			“No new information, but not the fault of the resource...”		36715		4 (2.8)	
	Resource did not focus on other substances or addictions			“You should expand this to include cannabis use too” “I would like to see other addiction issues discussed–particularly regarding technology”		34853 34429		3 (2.1)	
	Generic negative comment			“I just did not find it helpful.”		35629		2 (1.4)	
	Not a dislike			“I think a weekly sessions with a therapist would be helpful via zoom”		34368		2 (1.4)	
**Did you make any changes to your alcohol use based on the alcohol resource? (n=144)**									
	**Yes**								43 (29.9)
		Reduced drinking	“Yes, reduced alcohol consumption”		34811		27 (62.8)		
		Increased awareness of drinking habits	“It helped me realize sometimes I would grab a drink after a long day when my husband wasn’t home because I was lonely.”		34781		18 (41.9)		
		Replaced drinking with more helpful coping strategies	“...made healthier choices when I was having a bad day.”		34238		7 (16.3)		
	No			“No. I read the resource because I was curious (and like information) not because I think I have a problem with alcohol.”		36099		101 (70.1)	

#### Dislikes About the Resource

Most clients did not report disliking any aspect of the resource. Among clients who shared a dislike, the most common concern was that the resource was not relevant to their personal experience (eg, “I didn’t think it really applied, I’ve never really had issues with alcohol use outside a short stint in my late teens, early twenties.” [client ID 36202]). Other clients expressed concerns with the format or structure of the resource (eg, “I wish the PDF was fillable” [client ID 35890]) or felt that the resource did not provide them with any new information (eg, “mostly stuff I already knew” [client ID 34733]). Some clients found it challenging to have increased insight into the frequency of their alcohol consumption or severity of their alcohol concerns (eg, “It reminded me how much I’m binge drinking” [client ID 35312]). A small subgroup of clients thought that the resource should address other addictions or substance use (eg, “You should expand this to include cannabis use too” [client ID 34853]). Two clients responded with generic negative comments that did not fit into the other categories (eg, “I just did not find it helpful” [client ID 35629]). See Table 3 for a summary of clients’ dislikes regarding the alcohol resource.

#### Changes Made to Alcohol Use

In total, 3 types of changes emerged in clients’ responses, namely, reduced drinking (eg, “Yes I have cut down and now only will have a drink at a social function” [client ID 34667]), increased awareness of one’s drinking habits (eg, “It helped me realize sometimes I would grab a drink after a long day when my husband wasn’t home because I was lonely” [client ID 34781]), and replacing drinking with more helpful coping strategies (eg, “made healthier choices when having a bad day” [client ID 34238]).

## Discussion

### Principal Findings

This observational study investigated whether clients enrolled in an 8-week transdiagnostic ICBT course for depression and anxiety would review, benefit from, and positively evaluate an additional resource for addressing alcohol use. We also aimed to explore the demographic and clinically relevant variables associated with reviewing the alcohol resource. Across all groups, participants showed improvements in alcohol consumption, depression, and anxiety over time. Compared with nonreviewers, clients who accessed the resource were more likely to be older; men; and separated, divorced, or widowed. Furthermore, as expected, reviewers were more likely to consume more weekly drinks, report higher alcohol use difficulties, and have higher levels of hazardous drinking. Ratings and comments from resource reviewers indicated high satisfaction with the resource. The client ratings suggested that the resource was helpful and understandable, and most clients indicated that it was worth their time. Clients’ most liked aspects of the resource were that it was informative and that it assisted them in gaining insight into their drinking behavior. Resource reviewers did not differ from nonreviewers in any indices of overall treatment engagement (ie, course completion and website log-ins) or satisfaction with the ICBT course overall.

Interestingly, the prevalence of hazardous or harmful drinking based on AUDIT was slightly higher in this trial (242/1333, 18.15%) than in previous ICBT samples (160/1155, 13.85%) [5]. The elevated levels of hazardous drinking may be explained by the trial occurring during the COVID-19 pandemic, as 20% of Canadians who stayed home during the pandemic reported increased alcohol consumption [29]. Regardless, the high pretreatment hazardous alcohol use rates among clients are intriguing, given that the transdiagnostic ICBT program was intended to primarily address depression and anxiety symptoms. Clients scoring above the cutoff for hazardous drinking would have been eligible for another course offered by the Online Therapy Unit (ie, the Alcohol Change Course) [14], which focused primarily on alcohol use difficulties. It is possible that clients chose to enroll in the transdiagnostic ICBT course because of their desire to focus on depression and anxiety symptoms or because they had limited insight into their drinking concerns. Clients who were highly motivated to change their drinking behaviors may have enrolled in the Alcohol Change Course instead of the transdiagnostic ICBT course; therefore, although hazardous or harmful drinking was common in this trial, the clients who chose the transdiagnostic ICBT course may not have been motivated to change their drinking. As reductions in depression [30], depression, and anxiety [14] have been reported in trials of ICBT for alcohol use, these clients were likely to benefit from either course offered by the Online Therapy Unit.

Findings from this study are consistent with a randomized controlled trial that examined the inclusion of a brief web-based alcohol use intervention in conjunction with a web-based depression intervention [7]. Similar to that trial, we found no additional alcohol-related benefits associated with alcohol consumption, including a brief resource focused on reducing alcohol use. However, the inclusion of resources did not negatively impact clients’ engagement with the main intervention targeting depression and anxiety. Of note, while reviewing the resources was not associated with greater reductions in alcohol use, all transdiagnostic ICBT clients—regardless of whether they exhibited low-risk or hazardous drinking—showed decreased alcohol consumption scores, depression, and anxiety over time.

### Limitations and Future Directions

This study had several limitations that can help guide future research. The study was observational in nature and had no control group; therefore, causal conclusions about the impact of the resource cannot be made. The Alcohol Resource Evaluation Survey was administered at posttreatment, and responses were missing from approximately one-third of the clients. It is possible that some of the clients who did not respond to the Alcohol Resource Evaluation Survey actually reviewed the resource; however, because of their missing self-reports, we did not have information about their perceptions of the resource and whether the resource was helpful in reducing alcohol consumption by these clients. Future studies could include a resource evaluation survey at midtreatment to ensure higher response rates. Clients could also be asked about their alcohol consumption weekly to allow for tracking of changes in alcohol use over the span of the course. Moreover, it would be beneficial to use the Alcohol Timeline Followback [31] assessment method (ie, asking about consumption on each of the 7 preceding days) rather than the total weekly drinks to better assess the amount and pattern of drinking, which would allow for the assessment of heavy drinking days.

Only 10.8% (144/1333) of clients in this study reviewed the additional alcohol resource, including only 14.9% (36/242) of those with AUDIT scores indicating hazardous alcohol use. As the ICBT course included 19 additional resources, clients who may have benefited from alcohol may have prioritized other resources. Alternatively, clients who scored above the cutoff for hazardous drinking may not have perceived difficulties with their drinking patterns or they may have been focused on managing their symptoms of depression or anxiety before addressing any concerns related to alcohol. A limitation of the Alcohol Resource Evaluation Survey was that it did not ask clients to provide a reason for why they chose not to review alcohol resources. As such, future studies could include questions to better understand clients’ decisions not to review resources. Including a measure of motivation to change drinking behaviors could also be worthwhile, as this study only assessed need based on hazardous or harmful drinking, and it is not possible to determine how motivated these clients were to use resources or change their drinking behaviors. In the future, the main ICBT course content could include information regarding low-risk drinking guidelines and therapists could direct clients’ attention to the alcohol resource if they fall within the hazardous risk ranges for their drinking. Such methods could assist in increasing the uptake of alcohol resources, thereby facilitating more substantial opportunities for future research to elucidate the impact of reviewing resources in transdiagnostic ICBT. Therapists could recommend the resource at the beginning of the course based on clients’ pretreatment scores on the AUDIT or throughout the course based on clients’ concerns related to alcohol consumption. This study did not examine whether therapists recommend resource-predicted clients to review the resource, which could be an area of future study. It would also be worthwhile to ensure consistent follow-up from therapists regarding the resource after it has been recommended to clients.

Nearly one-fourth (326/1333, 24.46%) of clients reported never drinking alcohol on the pretreatment AUDIT; interestingly, 5.8% (19/326) of these clients reviewed the resource. In addition, almost half of the clients (662/1333, 49.66%) did not consume any drinks in the previous week at pretreatment, and 6.8% (45/662) of them reviewed the resource. This may have because of many reasons (eg, having a family member with alcohol use difficulties or having undisclosed or past problems with alcohol). Nonetheless, as only increases in drinking over time are possible for these groups, nondrinkers reviewing alcohol resources may have confounded the results, particularly for the mixed model analysis predicting changes in clients’ previous weekly drinks. We can also see echoes of further potential confounds within these nondrinkers’ qualitative feedback. For instance, the most common (12/33, 36%) dislike expressed by clients was that the resource was not relevant to their experience. As such, a further direction for research would be to randomly assign clients with alcohol use difficulties to (1) a transdiagnostic ICBT course targeting depression and anxiety or (2) the same transdiagnostic ICBT course plus an additional alcohol resource.

In terms of pretreatment characteristics, it is also important to highlight that most clients in this study identified as a woman and as White. These trends in mental health service use based on gender and ethnicity are not unique to ICBT [32,33]. Although acknowledging that biological sex differences are not inherently representative of differences based on gender identity, findings from epidemiological studies have suggested that alcohol use disorder is more common among male than female individuals [34] and that the relationship between alcohol use disorder and racial and ethnic groups is nuanced and influenced by historical and ongoing discrimination [35]. Therefore, the current sample may not be representative of the diverse population of individuals who experience alcohol-related difficulties. Future studies should aim to recruit a more diverse sample of clients.

### Strengths

To our knowledge, no previous studies on transdiagnostic ICBT for depression and anxiety have specifically examined the inclusion of a resource addressing alcohol use. As drinking difficulties are a prevalent concern among ICBT clients with depression and anxiety [5,6], this study makes an important contribution to the literature by examining the uptake and utility of a resource that can address this area of concern. The mixed methods (ie, both quantitative and qualitative data collections) approach is also a strength of this study. Furthermore, the study included a combination of objective and subjective measures of client engagement and perceptions of the resource, which can help inform changes to the content and presentation of the resource for future clinical research and practice.

### Conclusions

Consistent with previous research [5,6], nearly one-fifth of clients who enroll in transdiagnostic ICBT are identified as having problems with alcohol. A brief resource focused on alcohol use may be a way for some clients to address their concerns related to alcohol use while concurrently learning strategies to manage their symptoms of depression and anxiety. It appears that the resource is used more often by those who need it than by those who do not, and most reviewers are satisfied with the alcohol resource. Furthermore, some reviewers reported making changes to their alcohol use, such as increased awareness about drinking, reduced drinking, and the use of alternative strategies to cope instead of consuming alcohol. In general, ICBT was associated with improvements in alcohol use for all clients, and there was no added benefit in reviewing the resource. The minimal added benefit from reviewing the resource, especially for those with hazardous drinking, may reflect that more attention needs to be paid to ensuring that the resource is reviewed by those who need it.

## Abbreviations

AUDIT: Alcohol Use Disorder Identification Test
AUDIT-C: Alcohol Use Disorder Identification Test-Consumption
DUDIT: Drug Use Disorder Identification Test
GAD-7: Generalized Anxiety Disorder 7-item
ICBT: internet-delivered cognitive behavioral therapy
MCAR: missing completely at random
PHQ-9: Patient Health Questionnaire 9-item
QNR: questionnaire nonresponder
